# PHLS surveillance of antibiotic resistance, England and Wales: emerging resistance in Streptococcus pneumoniae.

**DOI:** 10.3201/eid0201.960108

**Published:** 1996

**Authors:** D C Speller, A P Johnson, B D Cookson, P Waight, R C George


### PHLS Surveillance of Antibiotic Resistance, England and Wales: Emerging Resistance in *Streptococcus pneumoniae*

To the Editor: The commentary, by Dr. M. S. Cetron and colleagues, on the action plan for drug-resistant *Streptococcus pneumoniae* (1) prompts us to describe the main system for surveillance of antibiotic resistance in use by the Public Health Laboratory Service (PHLS) in England and Wales and our recent results for resistance in *S. pneumoniae*.

Since 1974, the diagnostic laboratories in the PHLS network (53 in 1993-94) and increasing numbers of National Health Service and private laboratories have reported, on a voluntary basis, all bacterial isolations from blood or cerebrospinal fluid to the PHLS Communicable Disease Surveillance Centre. Since 1989, they have been asked to include their antimicrobial susceptibility test results on all isolates. In 1993, for example, antimicrobial susceptibility test results were received from 195 laboratories, on >28,000 nonduplicate isolates. Some data are sent as written entries on the report forms, but increasingly they are being transmitted electronically. Results may be analyzed according to region or reporting laboratory or by patient characteristics, such as age.

All laboratories do not test the same antimicrobial agents, but a nucleus set is tested by most laboratories for each species. Results are reported as "susceptible" (S), "resistant" (R) or "intermediate" (I). Although the methods are not standardized, external quality assurance is provided by the UK National External Quality Assessment Scheme (2), to which almost all laboratories subscribe. In addition, many laboratories refer isolates that show particularly critical resistance traits (such as ß-lactam resistance in *S. pneumoniae*, or glycopeptide resistance in *Enterococcus* species) to the PHLS Antibiotic Reference Unit (ARU) for determination of minimum inhibitory concentrations (MICs); these can often be matched against the submitted results. Occasional prevalence surveys in the PHLS network, with testing of isolates in the ARU, act as a further monitoring measure.

The application of the system can be seen in the recently published results of 6 years' surveillance of resistance amongst isolates of *S. pneumoniae* causing bacteremia or meningitis (3,4). There has been a statistically significant trend to increased resistance to penicillin (from 0.3% in 1989 to 2.5% in 1994), although these are low percentages in comparison with those seen in many other countries (5). Moreover, a considerable increase has been observed in resistance to erythromycin (from 3.3% in 1989 to 11.2% in 1994). These figures are based on susceptibility testing of more than 2,500 isolates in each of the 6 years. In 1993, resistance to penicillin and erythromycin was significantly more common amongst pneumococci from bacteremia and meningitis in the younger age groups (≤ 9 years). A significant rise during the 6 years was also seen in trimethoprim resistance, but no significant change was observed in resistance to tetracycline or chloramphenicol.

The resistance totals include isolates reported as resistant (R), and as intermediate (I), as we cannot be sure of the basis for this discrimination in the diagnostic laboratories. The proportion of isolates reported as I is very small, with the exception of antimicrobial agents for which many strains have MICs near the breakpoint defined to separate sensitive and resistant strains. In the case of *S. pneumoniae*, this applied to trimethoprim (6.3% of isolates reported as I in 1993); < 0.5% of isolates were reported as I to other antimicrobial agents.

The results of the National External Quality Assessment Scheme exercises have shown acceptable proficiency. For example, in the detection of penicillin resistance, in five distributions of pneumococcal strains that require an MIC of 0.25 mg/l, 74% to 90% of laboratories obtained correct results; in six distributions of strains that require an MIC of 1.0 mg/l. 95% to 99% of laboratories did so (J.J.S. Snell, pers. comm.). The isolates included in the analysis that were also tested in the ARU gave closely similar results: for example, of 86 pneumococcal isolates tested for susceptibility to penicillin in early 1995, 82 (95%) gave the same result in the sender's laboratory and in the ARU. A survey of resistance in pneumococci (from all sites) conducted in March l995 with MIC determination by the ARU (unpublished) showed similar proportions of resistant strains.

These observations demonstrate that the results of susceptibility tests undertaken for the management of individual patients may be compiled and analyzed for surveillance purposes. Duplicate isolates from the same infection episode should not be included, and satisfactory quality assurance should be undertaken. Increasing use of computers and networking among the clinical laboratories should facilitate the process of data collection.

**David C.E. Speller, Alan P. Johnson, Barry D. Cookson, Pauline Waight,* Robert C. George**
Central Public Health Laboratory and *Communicable Disease Surveillance Centre, 61 Colindale Avenue, London NW9 5HT, UK

### References

1. Cetron MS, Jernigan DB, Breiman RF, DSRP Working Group. Action plan for drug-resistant *Streptococcus pneumoniae*. Emerging Infectious Diseases 1995;1:64-5.
2. Snell JJS, de Mello JV, Gardner PS. The United Kingdom national microbiological quality assessment scheme. J Clin Pathol 1982;35:82-93.
3. Aszkenazy OM, George RC, Begg NT. Pneumococcal bacteraemia and meningitis in England and Wales 1982 to 1992. CDR Review 1995;5:R45-R50.
4. Antibiotic resistance in Streptococcus pneumonia 1993 and 1994. Commun Dis Rep 1995;5:187-8.
5. Friedland IR, McCracken GH. Management of infections caused by antibiotic-resistant *Streptococcus pneumoniae*. N Engl J Med 1994;331;377-82.

